# Supplementary figures and images for: Human cytomegalovirus long noncoding RNA4.9 regulates viral DNA replication
Source: PLoS Pathog. 2020 Apr 15;16(4):e1008390. doi: 10.1371/journal.ppat.1008390 (PMC7185721; doi:10.1371/journal.ppat.1008390)

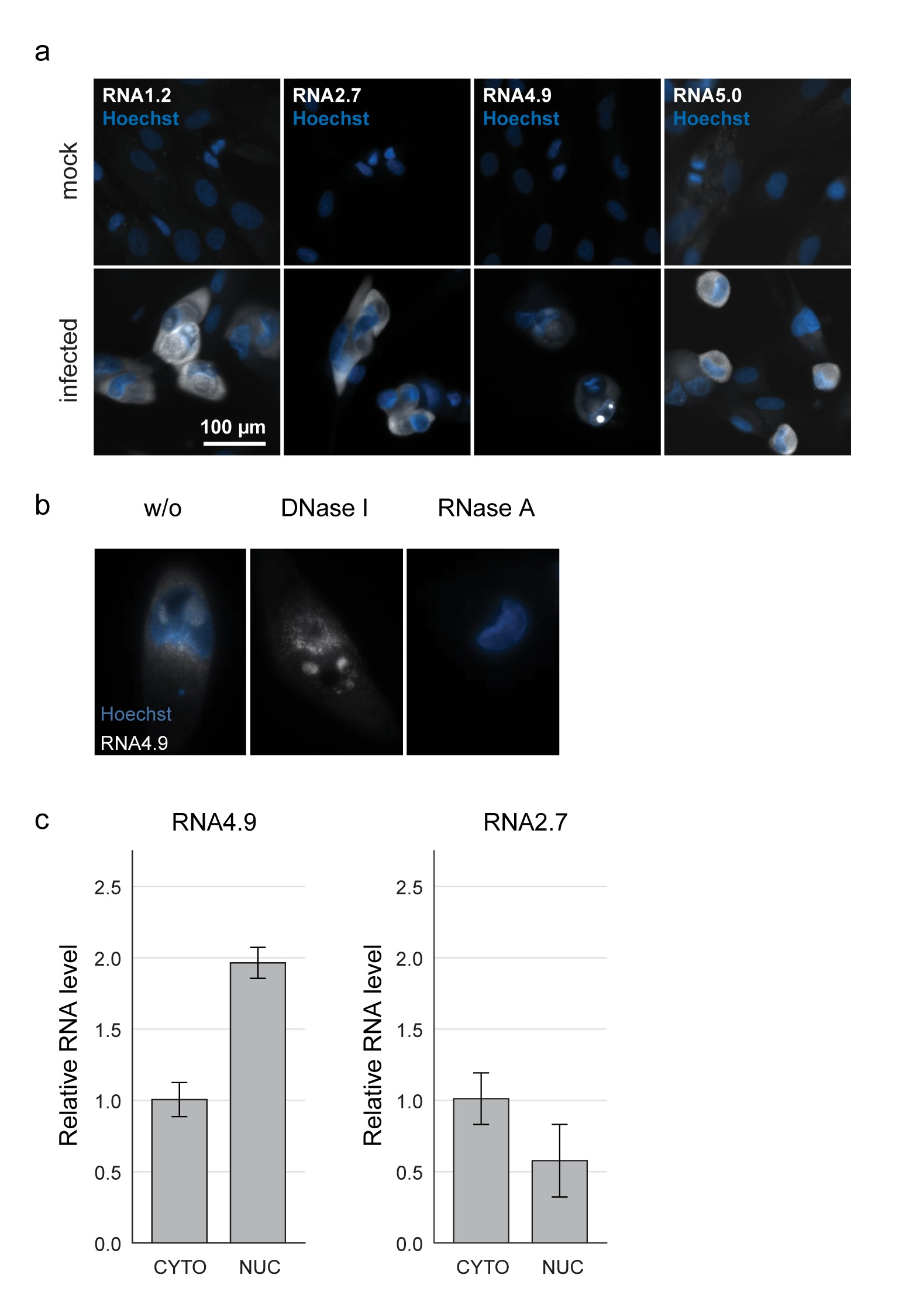

Supplement: S1 Fig — a) Mock and HCMV Merlin strain-infected fibroblasts at 48 hpi (MOI = 5) were stained using fluorescent probes (white) against indicated HCMV lncRNAs. b) RNA4.9 was detected by RNA-FISH using fluorescent probes (white) in HCMV Merlin strain-infected fibroblasts at 48 hpi (MOI = 5). Cells were untreated, pretreated with DNase I or RNase A as indicated. a-b) Nuclei were counterstained with Hoechst (blue). c) At 48 hpi (MOI = 3), infected fibroblasts were fractionated into cytosolic (CYTO) and nuclear (NUC) fractions. Relative RNA4.9 and RNA2.7 levels were quantified using RT-qPCR and normalized to the cytosolic fraction and the cellular transcript ANXA5. (TIF) [file ppat.1008390.s001.tif]

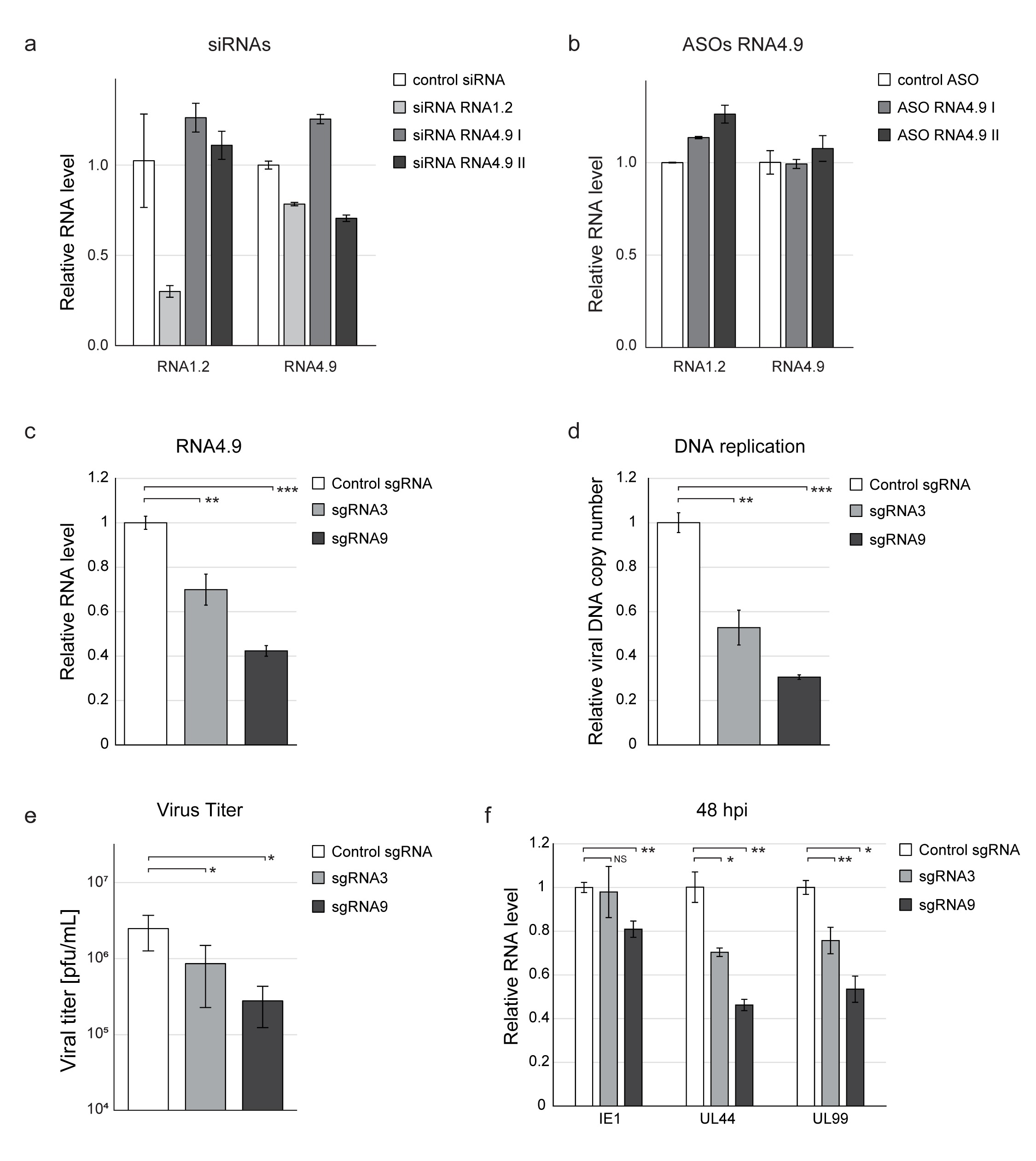

Supplement: S2 Fig — a) Fibroblasts transfected either with control siRNA or siRNAs targeting RNA1.2 and RNA4.9, respectively, were infected with HCMV Merlin strain (MOI = 1). Relative levels of RNA1.2 and RNA4.9 were quantified using RT-qPCR at 48 hpi, and normalized to the cellular transcript ANXA5. b) Fibroblasts transfected either with control ASOs or ASOs against RNA4.9, were infected with HCMV Merlin strain (MOI = 1). Relative levels of RNA1.2 and RNA4.9 were quantified using RT-qPCR at 48 hpi, and normalized to the cellular transcript ANXA5. c-f) Fibroblasts expressing dCAS9 and either a control sgRNA or one of two different sgRNAs targeting the RNA4.9 promoter (sgRNA3 and sgRNA9) were infected with HCMV Merlin strain (MOI = 0.1). c) Relative RNA4.9 levels were quantified using RT-qPCR at 48 hpi and normalized to the human transcript ANXA5. d) Relative viral DNA levels were quantified using qPCR at 48 hpi using UL55 primers, and normalized to the cellular gene B2M. e) Viral titers were measured 5 days post infection (dpi) by TCID50. f) Relative levels of the UL123 (IE1), UL44 and UL99 transcripts were quantified using RT-qPCR at 48 hpi and normalized to the cellular ANXA5 transcript. c-f) Values and error bars represent the average and SD of triplicates. A representative analysis of two independent experiments is shown. Two-sided t-test was applied (***p-value<0.001, **p-value<0.01, *p-value<0.05, NS, not significant). (TIF) [file ppat.1008390.s002.tif]

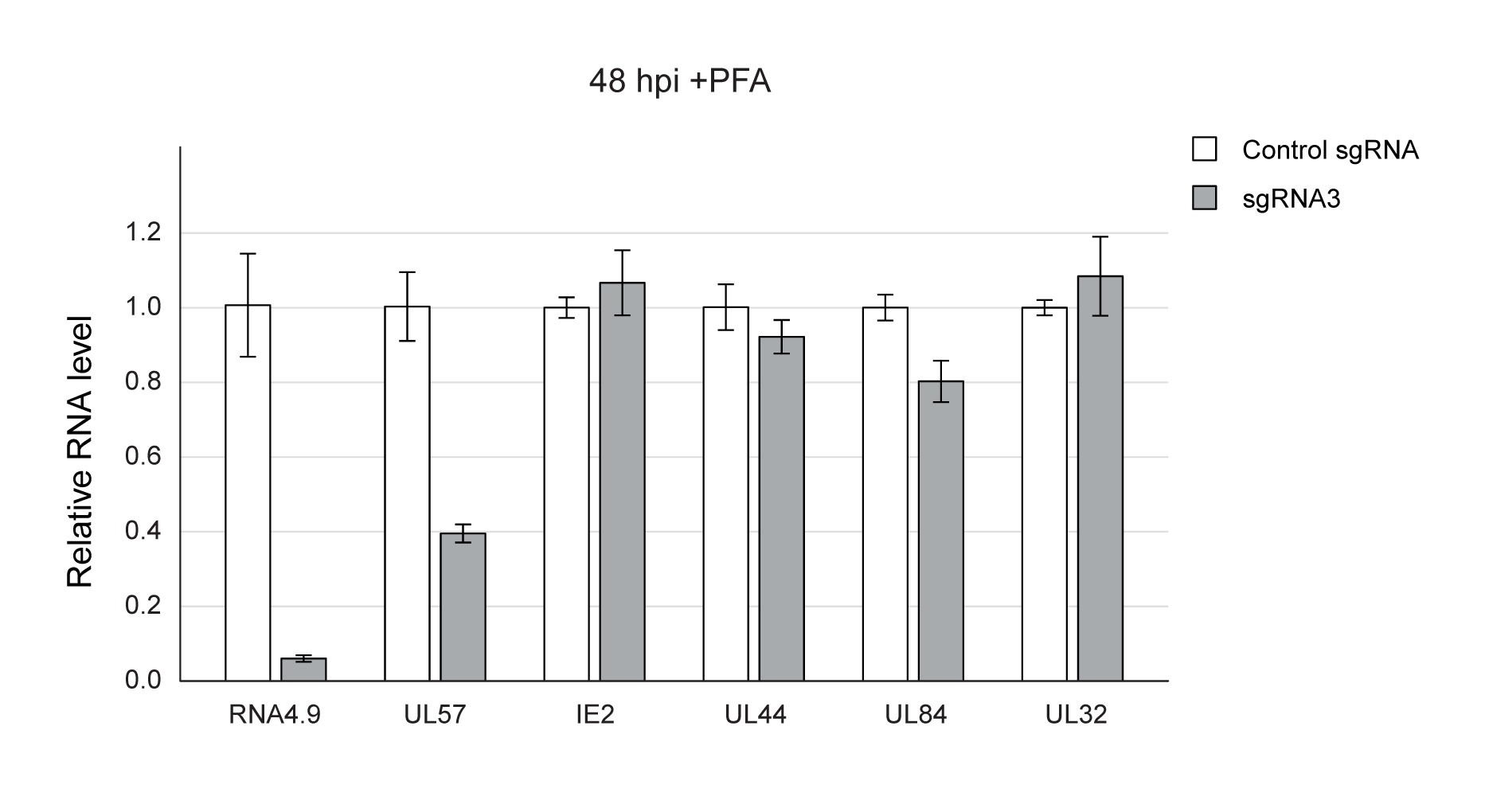

Supplement: S3 Fig — Fibroblasts expressing CAS9 and either a control sgRNA or a sgRNA targeting the RNA4.9 promoter (sgRNA3) were infected with HCMV Merlin strain (MOI = 2) and treated with PFA. Relative levels of the indicated viral genes, including RNA4.9, were quantified using RT-qPCR at 48 hpi and normalized to the cellular transcript ANXA5. (TIF) [file ppat.1008390.s003.tif]

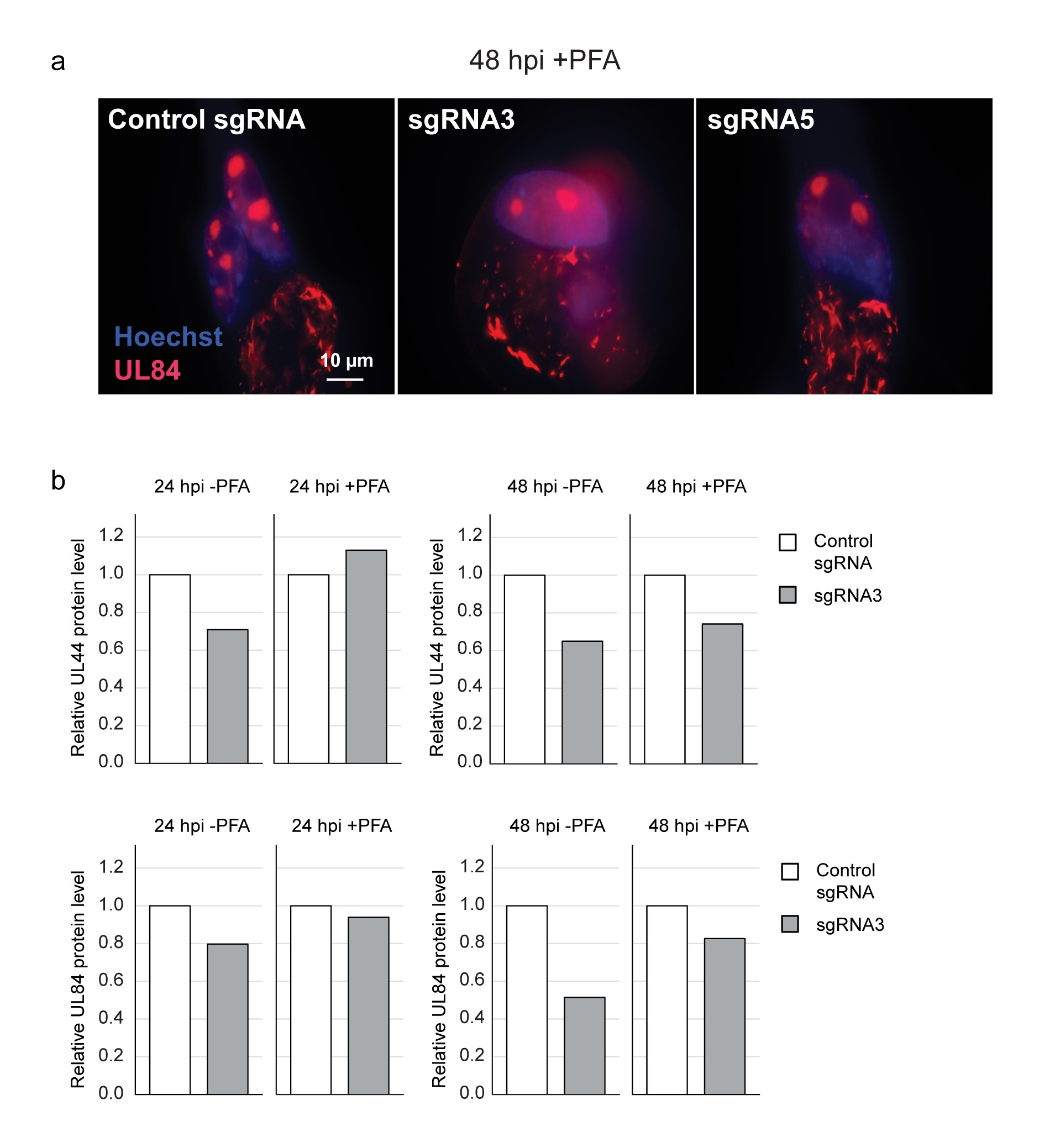

Supplement: S4 Fig — Fibroblasts expressing CAS9 and either a control sgRNA or a sgRNA targeting RNA4.9 (sgRNA3 or sgRNA5, as indicated) were infected with HCMV Merlin strain (MOI = 3). a) UL84 (red) was detected using IF at 48 hpi in the presence of PFA. b) Quantification of UL44 and UL84 protein levels from the immunoblot analysis in (Fig 4D), normalized to the levels of GAPDH. (TIF) [file ppat.1008390.s004.tif]

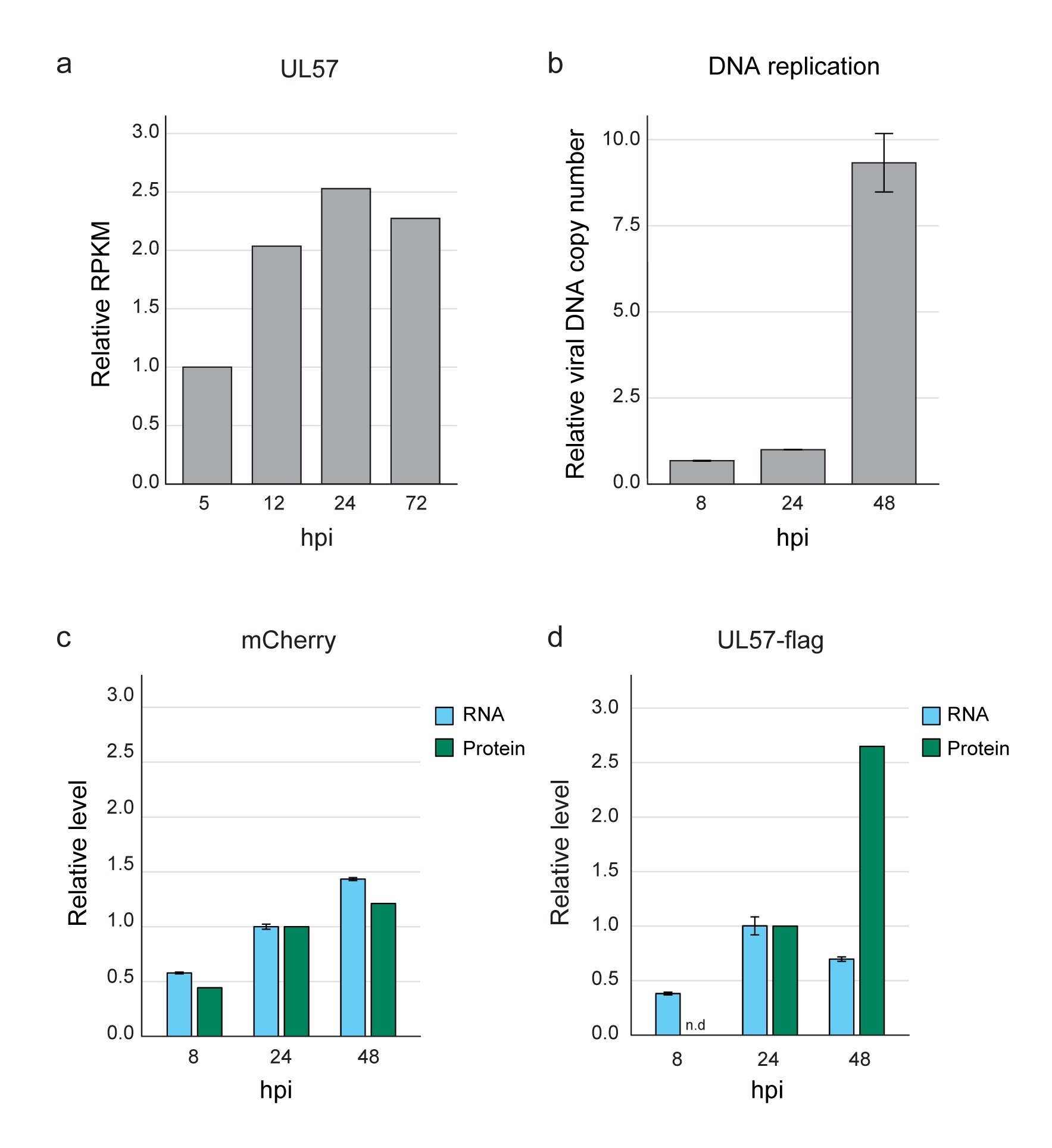

Supplement: S5 Fig — a) Relative expression of UL57 transcript during HCMV infection as measured by RNA-seq [24]. b) Fibroblasts were infected with HCMV Merlin strain (MOI = 2) and harvested at the indicated time points post infection. Relative viral DNA levels were quantified using qPCR at the indicated time points, using UL44 primers and normalized to the cellular gene B2M. (TIF) [file ppat.1008390.s005.tif]

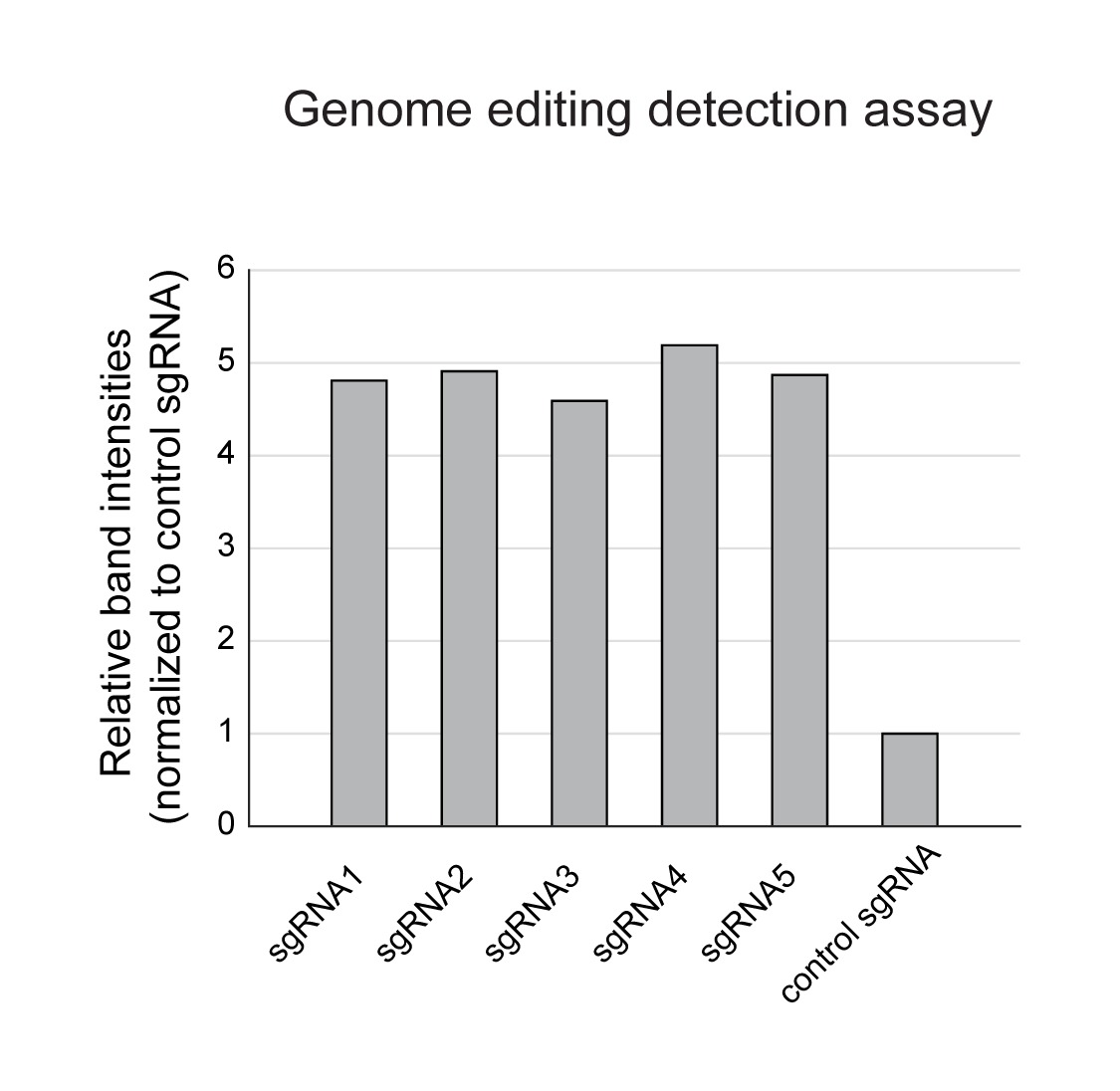

Supplement: S6 Fig — Fibroblasts expressing CRISPR-Cas9 and five different sgRNAs targeting the RNA4.9 TSS region or a control sgRNA were infected with HCMV Merlin strain (MOI = 3), treated with PFA and harvested at 48 hpi. A T7 endonuclease I mismatch cleavage assay was conducted to estimate the genome editing efficiency of the RNA4.9 loci. The relative quantification of band intensities (which indicates a mutated sequence) is presented, normalized to the signal obtained using the control sgRNA. (TIF) [file ppat.1008390.s006.tif]

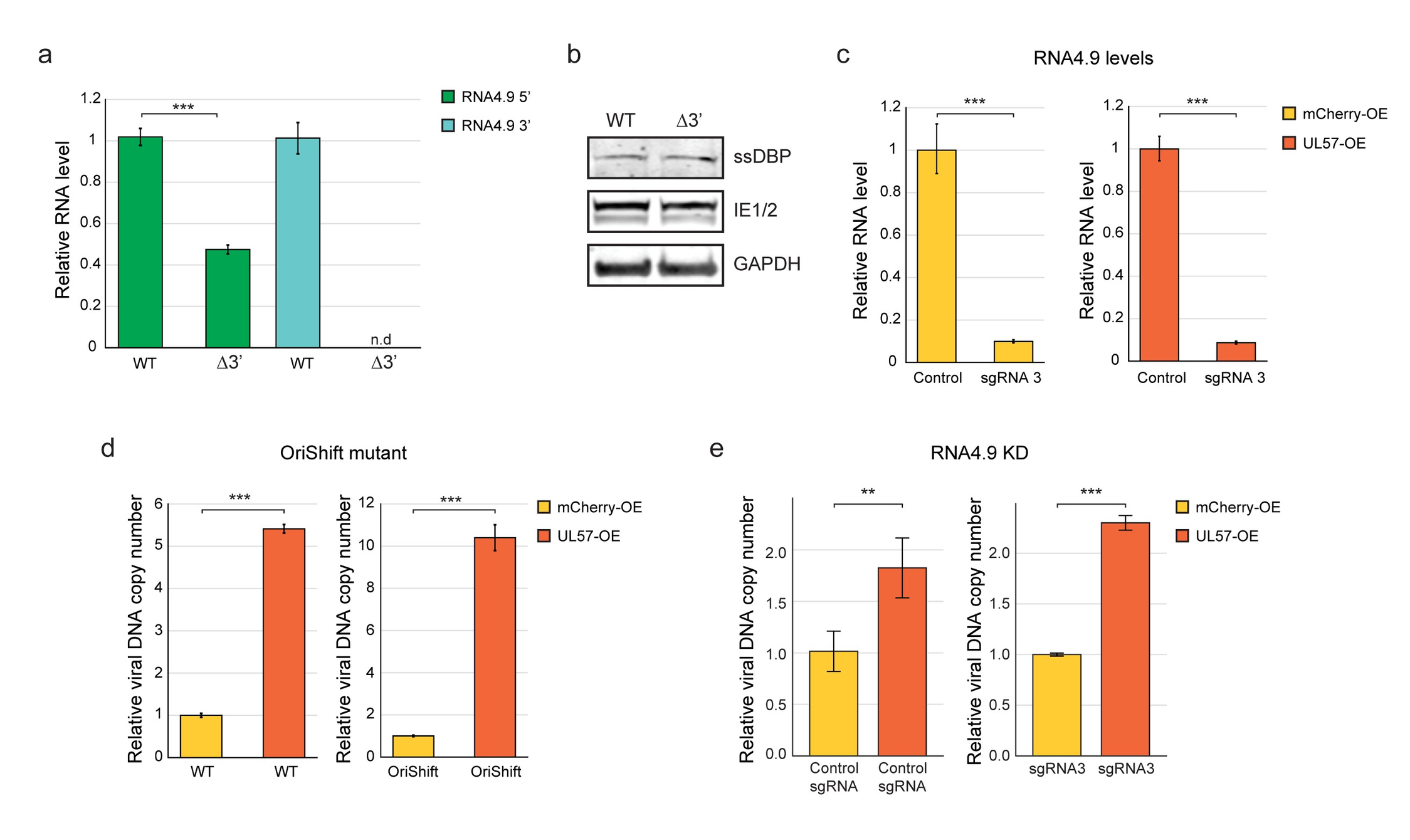

Supplement: S7 Fig — a-b) Fibroblasts were infected with the Merlin Δ3’ mutant or the parental strain (MOI = 1), treated with PFA, and harvested at 48 hpi. a) Relative RNA4.9 5’ and 3’ levels were quantified using RT-qPCR and normalized to the cellular ANXA5 transcript. b) ssDBP and IE1/2 were detected by immunoblot analysis. Human GAPDH was used as a loading control. c-e) Fibroblasts expressing mCherry as control (yellow) or ssDBP (orange) were infected, as indicated (MOI = 1), and harvested at 48 hpi. c) Relative RNA4.9 levels were quantified using RT-qPCR and normalized to the cellular ANXA5 transcript in fibroblasts expressing CRISPR-Cas9 and a sgRNA targeting the RNA4.9 TSS region (sgRNA3)or a control sgRNA and infected with the HCMV Merlin strain. d) Fibroblasts were infected with the OriShift mutant virus or the parental strain (AD169). Relative viral DNA levels were quantified by qPCR using UL44 primers, and normalized to the cellular gene B2M. e) Fibroblasts expressing CAS9 and either a control sgRNA or a sgRNA targeting RNA4.9 TSS region (sgRNA3) were infected with HCMV Merlin strain. Relative viral DNA levels were quantified using qPCR and UL44 primers, and normalized to the cellular gene B2M. a, c-e) Values and error bars represent the average and SD of triplicates. A representative analysis of two independent experiments is shown. Two-sided t-test was applied (***p-value<0.001, **p-value<0.01, n.d, not detected). (TIF) [file ppat.1008390.s007.tif]

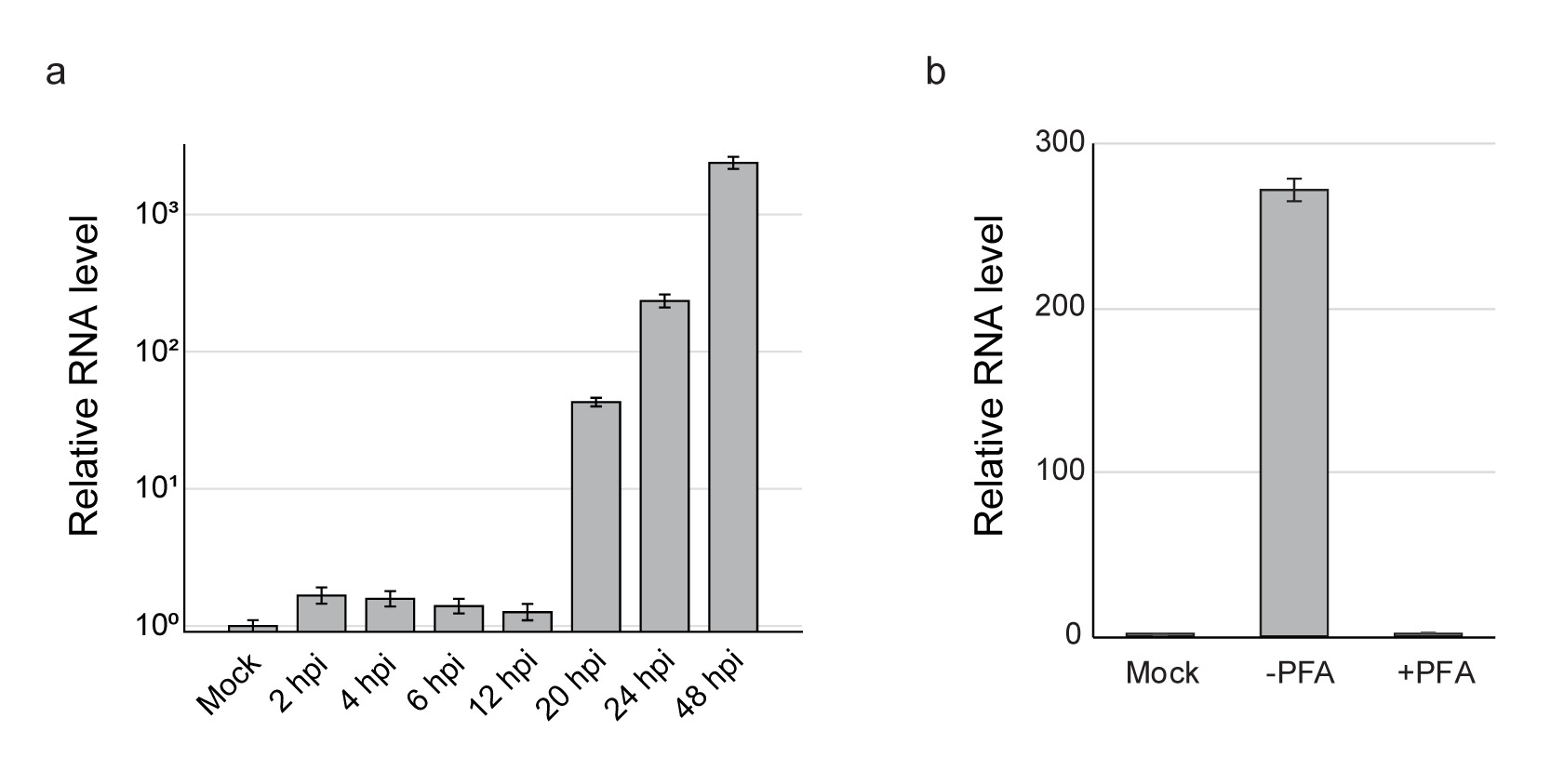

Supplement: S8 Fig — a) Relative RNA1.6 transcript levels in MCMV Smith strain-infected MEFs (MOI = 1) were quantified by RT-qPCR at indicated time points post infection. RNA levels were normalized to the mouse GAPDH transcript. b) MEFs were either infected with MCMV Smith strain (MOI = 1) or left uninfected, and the infected cells were either treated or untreated with PFA. Relative RNA1.6 levels were quantified by RT-qPCR at 20 hpi. RNA levels were normalized to the mouse 18S rRNA. a-b) Values and error bars represent the average and SD of triplicates. A representative analysis of two independent experiments is shown. (TIF) [file ppat.1008390.s008.tif]
