# Supplementary material for: Human cytomegalovirus long noncoding RNA4.9 regulates viral DNA replication
Source: PLoS Pathog. 2020 Apr 15;16(4):e1008390. doi: 10.1371/journal.ppat.1008390 (PMC7185721; doi:10.1371/journal.ppat.1008390)
Supplement: Attachment — Submitted filename: point_by_point_response_final.docx [file ppat.1008390.s011.docx]

**Point by Point response to the reviewers comments**

We would like to thank the reviewers for their time and thoughtful comments, which we feel helped us to substantially improve our manuscript.


Reviewer #1: none.

Reviewer #2: Major concern
1. Their approach to knock-down the gene of interest using WT Cas9 will lead to complex and varied mutations and possibly indirect host DNA damage responses.
The authors have used gene-editing with wt Cas9 to generate dsDNA breaks in the CMV genomes. This is not an appropriate way to transiently knock-down a specific gene of interest in the context of infection (why not use siRNA?).

Our first attempts to characterize RNA4.9 functions were using siRNAs. While we were able to knock down the expression of RNA1.2 expression (An hcmv-lncRNA that is mostly cytoplasmic) we did not observe any significant reduction in RNA4.9 expression using siRNA, probably due to the fact that the RNAi machinery is mainly located in the cytoplasm and the access to nuclear-retained lncRNAs is limited (Zeng and Cullen, 2002). This data was now added to the manuscript (new Figure S2a). We also attempted to use chemically modified chimeric DNA antisense oligonucleotides (ASO) that are often used to deplete nuclear RNAs, but also the ASOs we tested did not lead to significant reduction in RNA4.9 expression (new Figure S2b), in accordance with reports showing ASOs are often inefficient (Ramilowski et al., 2019).

This type of approach is especially troublesome in the area of the genome that houses both the origin of lytic replication and the regulatory region of the DNA replication proteins that is focused on. A break in this area can cause non-specific host DNA damage responses, loss-of-function of the origin and disruption of the normal architecture of the genome that mediates transcription of genes in that area. Given the complexity of this region and the lack of characterization of the ensuing genome edits, it is impossible to interpret the downstream effects. The use of multiple gRNAs and seeing disparate effects merely indicates the efficiency of edits and damaged genomes differs. Their control gRNA will likely not cut at all- they might want to target an innocuous region (this is obviously hard to do). They do not sequence the edited genomes. Cas9 systems lead to a spectrum of mutations. Conclusions about the direct effect of loss of the lncRNA4.9 can not be made using this approach. If the authors wish to use this method to edit the virus, they might purify and characterize the mutants prior to use in infection experiments to better ensure that only lncRNA4.9 is directly impacted. This will be very difficult to do. More refined mutations such as disruption of initiation or insertion of a transcriptional stop would seem much cleaner.

We appreciate the reviewer concerns about indirect effects generated by the CRISPR-Cas9 cutting. We have included in the manuscript a set of experiments that are aimed to address this possibility. We conducted RNA-seq on cells in which RNA4.9 is targeted by CRISPR-Cas9 and we did not observe substantial changes in any gene besides RNA4.9 (Figure 3 and Table S1). Additionally, we tested the efficiency of Cas9 induced editing by six sgRNAs all targeting RNA4.9 TSS and although we did not observe substantial differences in their induced editing efficiency (Figure S6), the sgRNAs that hindered RNA4.9 expression also resulted in reduction in the ssDBP. Still we agree with the reviewer that these experiment do not unambiguously preclude indirect effects that are caused by the cutting and we now explicitly discuss this possibility (lines 324-327 and 350-356). Nevertheless the two main conclusions of our work ; 1. That RNA4.9 plays a role in regulating HCMV DNA replication and 2. that the ssDBP levels are coupled to the activity of *oriLyt*; are substantiated by additional evidence that is independent of CRISPR-Cas9 cutting. These experiments are listed below and are also discussed in the manuscript (lines 328-345).

Several lines of evidence indicate that RNA4.9 is involved in viral DNA replication; First, RNA4.9 is transcribed early in infection within the *oriLyt,* it generates DNA-RNA base pairing that in other herpesviruses were shown to be important for origin unwinding and it is localized in the viral replication compartment. Although none of these observation directly prove RNA4.9 is involved in viral DNA replication they are all novel and all strongly support this hypothesis. Second, the use of RNA4.9-Cas9 KD resulted in substantial and specific reduction in RNA4.9 expression that coincided with reduction in viral DNA replication. Although we cannot preclude the possibility that the physical DNA cleavage generated by the Cas9 system is responsible for the observed phenotypes, previous work (van Diemen et al., 2016; Hein and Weissman, 2019) and our data illustrate that cleavage of HCMV DNA has only a modest impact on genes proximal to the cut site (by RNA-seq) and minimal impact on HCMV replication. In addition, the observation that much of the growth defect caused by RNA4.9-Cas9 KD could be rescued by over expression of ssDBP, further indicates that there is no major damage in the *OriLyt* itself. Third, RNA4.9 KD using CRISPRi, in which RNA4.9 transcription is blocked without causing DNA breaks or mutations, also results in a reduction in viral DNA replication. Finally, we now added data using a new viral mutant we generated in which RNA4.9 TATA box was deleted (ΔTATA) and we show this mutant also shows reduction in viral DNA replication. Altogether, these findings strongly support a direct involvement of RNA4.9 in viral DNA replication.

Our results also show that ssDBP levels are tightly coupled to the *oriLyt* activity; Following both CRISPR-Cas9 targeting and genetic relocation of *oriLyt,* ssDBP levels were substantially reduced but there were no major changes in UL57 transcript levels. In addition, during infection the ssDBP could be detected only when viral DNA replication started and only at the replication compartment (new Figure 5c). Finally, ectopically expressed ssDBP could be seen only when cells were infected and only at time points in which DNA replication started, despite the transgene being expressed under an exogenous promoter.

We now clearly state in several places that we cannot preclude indirect effects of CRISPR-Cas9 cutting and we explicitly discuss the limitations of our experiments. We hope the reviewer will appreciate that our findings, which now include additional data and answer most of the reviewers concerns, do shed light on the role of RNA4.9 in HCMV replication and the complex regulation of the oriLyt activity.

The use of a mutant Cas9 that impairs transcription initiation is a more reasonable approach and there is one supplemental figure demonstrating an impairment in lncRNA expression and replication. How does the alteration of transcription in this region impact the ‘firing’ of the lytic origins? Rescue by ectopic lncRNA4.9 expression should be demonstrated.

Measuring the firing of the origin of replication is not a trivial task (especially since we are interested in measuring the unwinding and not the firing itself) and out of the scope of our lab expertise. We did clone and ectopically express RNA4.9 but the levels of expression we obtained were 1000-fold lower than the expression we measured during infection (see Figure below), making any complementation assay unrealistic. Moreover, since it is possible that the role of RNA4.9 in viral DNA replication regulation involves its transcription in *cis*, its ectopic expression may not rescue the phenotype.

We believe that altogether, the results presented in the manuscript strongly support a direct role of RNA4.9 in the regulation of viral DNA replication.

RNA4.9 expression levels were measured by RT qPCR in fibroblasts ectopically expressing RNA4.9 (I and II represent two independent transductions), in fibroblasts infected with HCMV Merlin strain (MOI = 3) and uninfected cells.

2. Experimental reproducibility and rigor throughout the paper.
In general, the MOI varies within each figure and across nearly all figures in the paper. This makes integration of RNA, DNA, Protein, and IFA data very difficult. Specific concerns with each figure are described below.

Figure 1.
1A appears to be previously published data. What was the cell type and MOI? How do these levels relate to coding transcripts typical of IE, E, L.

We added all the requested details to the figure legend. We revised figure 1a to include a representative viral gene of each kinetic class (see Figure below), however the figure in the manuscript presents the median viral gene expression. This presentation emphasizes the extremely high expression of the viral lncRNAs while keeping the figure easily accessible.

Expression of viral genes (IE1, immediate early; UL44, early; UL32, late) and viral lncRNAs during HCMV infection as measured by RNA-seq.

1B. What is the nuclear to cytoplasmic ratio? Why not show dual color as in 1C. Is there a software-based enumeration of this data so we can learn from hundreds of cells, not single cell staining?

To support the findings presented in this figure using a more quantitative measurement, we performed subcellular fractionation and analyzed RNA 4.9 expression in the nuclear and cytoplasmic fractions. In agreement with our FISH staining, RNA4.9 was enriched in the nuclear fraction, whereas RNA2.7 was mostly cytosolic (new Figure S1c).

1B-1C. Where is the mock control? There is no direct demonstration of viral DNA replication compartments

As requested by the reviewer we now also show the control mock cells that we have used in these FISH experiments (new Figure S1a). In addition to further demonstrate these foci indeed represent sites in which viral DNA replication occur we labeled nascent viral DNA in infected cells with ethynyl-2′-deoxyuridine (EdU) and visualized it using the “Click” chemistry. This new data illustrates that RNA 4.9 indeed localizes in the viral DNA replication compartment (new Figure 1d)

Figure 2.
2A. There seem to be disparities in the impact on RNA, DNA, virus production between sgRNA3 and 5. The impact on gene expression seems to be greater with sgRNA3 at 24 hpi and with sgRNA5 at 48 hpi. Why was sgRNA 3 used moving forward?

We have conducted many experiments using these two sgRNAs and no substantial differences between the guides were found. We are pasting an example of such experiment below. Small differences between experiments could be explained by differences in the transduction efficiency of the sgRNAs into fibroblasts.

Viral gene expression was measured by RT qPCR in fibroblasts expressing Cas9 and either a control sgRNA or one of two different sgRNAs targeting the RNA4.9 promoter (sgRNA3 and sgRNA5). Viral genes were normalized to the human transcript ANXA5.

Figure 3.
3A. This was based on one experiment. Why was a low MOI used for this? Why weren’t more genes impacted.

The presented RNA-seq experiment was performed on two biological replicates for both the control and RNA4.9 sgRNAs. We now indicate this information more clearly in the figure legend and we add SD that indicates the noise of our replicate measurement. This experiment was done in low MOI by mistake. To further show that similar results are obtained in higher MOI of infection, we performed real-time PCR analysis of cells infected in MOI=2. This experiment shows the same result as seen in the RNA-seq, that there is no substantial change in viral gene expression when RNA4.9 expression is knocked down using CRSIPR-Cas9 (new Figure S3). This experiment was done with PFA, so that only effects of RNA4.9 KD on gene expression, which are not through its effect on DNA replication, will be apparent. None of the genes besides RNA4.9 itself are impacted meaning that there are no direct effect of RNA4.9 KD on viral gene expression.

3B. The normalization of this data is really difficult to understand. There is no data regarding the OriLyt.

The figure legend has been revised so the normalization will be clearer. The graph shows the enrichment of viral lncRNAs genomic loci (green) and an R-loop-forming cellular gene (APOE, blue; positive control) relative to the immunoprecipitation with an isotype control using quantification by qPCR. Fold-enrichment was calculated relative to a non-R-loop-forming cellular gene (SLC22A1). Relative enrichment of 1 represents no enrichment (labeled now by grey dashed line).

Figure 4.
A and b. 24 hpi is shown without PFA and the 48 hr timepoint is with PFA. This is a strange comparison.

PFA abolishes viral replication compartments, does this change the nuclear localization of RNA4.9?

Since we were interested to look at the direct effect of the RNA4.9-Cas9 KD (not due to reduction of DNA template) on the ssDBP, we used PFA to inhibit replication at 48hpi, when replication is extensive, while this is less critical at 24hpi when replication only starts. Nevertheless, for completeness we now also add the data from 48hpi (new Figure 4b).

We also added data illustrating that RNA4.9 localizes to nuclear foci independent of DNA replication as we can detect it even when DNA replication is inhibited with PFA (new Figure 2b).

C. Isn’t it expected that PFA or anything that abrogates lytic replication will reduce viral gene expression?

The reviewer is correct and this is indeed shown in Figure 4d; the addition of PFA leads to reduction in IE2, UL84, UL44 and UL57. However, this experiment also shows that with PFA (neutralizing any effects that stem from reduction in DNA replication) only UL57 expression is affected.

D. no error bars

The quantification that is presented in this figure (new Figure 4e) is of the western blot that is presented in the figure together with the RNA that was extracted from the same cells. Since this is quantification of this parallel western blot there is no SD. However, this experiment was repeated two independent time and gave similar results (substantial change in the protein but minimal effect on the RNA).

E. This should be provided with the exact coordinates of the sgRNAs used earlier in the manuscript.

This information was now added to figure legend

Figure 5.
How many experimental replicates does this represent?
This experiment was repeated in two independent experiments

It is difficult to compare transcripts to protein. Such correlations are not very valuable and no viral DNA replication data is provided for reference. How is the quantitation of transcripts and proteins from an overexpressed gene informative?

We agree it is difficult to compare protein and transcript levels. What this figure and the corresponding supplementary figure (Figure S5) show is that by both RNA-seq and real-time PCR UL57 transcript is expressed from an early time point post infection and does not dramatically change when viral DNA replication starts (as one would expect from an early gene). However, the protein itself could be detected only after DNA replication starts (around 24hpi in HCMV). The same point is illustrated using an overexpressed transgene (although the transcript is there the protein is identified only when viral replication starts).

To strengthen this point we now also added microscopy data showing that although RNA4.9 could be detected early in infection (12hpi) we could detect UL57 only at time points in which viral DNA replication starts and only next to the replication compartment (new Figure 5c)

Figure 6.
Different sgRNAs in the lncRNA4.9 region have differential editing of the genome that leads to dsDNA breaks. This does not show a direct effect of one transcript to another.

Although the five different sgRNAs have different efficiency of RNA4.9 KD, we show in figure S6 that this is likely not due to differences in cleavage efficiency. A possible explanation is that mutations in different sites of the TSS have different effects on transcription from that TSS.

Figure 7.
7D. This is an unusual mutant. Does the previous publication describe a replication defect at this MOI?

The previous publication showed that this mutated virus (named in the original paper, pHG-6 ) has growth defect in both low and high MOI but they report there are no major differences in viral DNA replication using slot blotting with HCMV probe (Figure 6 in Borst *et al*. 2005).

7F. Does ssDNA overexpression increase DNA copy number?
F and G. What was the statistical test used? The impact of ssDBP is very slight.
B-F, if you overexpress full-length lncRNA4.9, do you rescue ssDBP, replication and infectious virus production back to WT levels?

As shown in Figure S7d and S7e and as discussed in the text, over expression of UL57 significantly increase viral DNA replication in both WT and mutated setting.

Note that the scale in Figures 7f and 7g is log scale. These differences are reproducible and were tested using two-way ANOVA on biological replicates. As indicated above the levels of RNA4.9 expression we obtained by ectopically expressing RNA4.9 are far lower than the expression we measured during lytic infection, making complementation experiments not feasible. Moreover, since our results indicate that probably at least part of RNA4.9 effects are in *cis,* interpretation of a complementation assay will be hard if not impossible.

Figure 8.
A Where do the sgRNA bind and edit in MCMV? What are those edits?

We now added labeling that indicates the location of the sgRNA target sites to figure8a (the PAM sites are now also specified in the figure legend). We did not characterize the edits that are generated by these sgRNAs as the characterization of the murine homologue is out of the scope of our current manuscript.

3. The model is not supported by the data.
There are no experiments using proteasome inhibitors to support premise of ssDNABP instability. No data for direct interaction of lncRNA4.9 with the oriLyt. No data for impact on nascent DNA replication or unwinding at oriLyt.

What is presented in Figure 9 is a **proposed** model that can potentially explain the results we present in the manuscript. Our data strongly indicates that RNA4.9 is regulating viral DNA replication (potentially through R-loop formation). We further show that the levels of ssDBP are coupled to the activity of *oriLyt* and our results suggest this is probably due to reduced stability in the absence of origin unwinding (although we do not show this directly). The parts that are more speculative are marked by dotted lines, and in the revised figure we also added question marks (so it will be clear these are not established links). We feel putting together a model helps to convey the main findings presented and place them in a wider context.

Reviewer #3:

The timing of the onset of DNA replication and the expression of RNA4.9 needs to be clarified. DNA replication begins around 24 hrs post infection. The expression kinetics of RNA4.9 needs to be confirmed at earlier time points before DNA replication begins in order to determine the role of RNA4.9 in DNA synthesis. The question is, what is the level of RNA4.9 between 24 and 36 hrs? The relationship between the levels of RNA4.9 and amount of newly synthesized DNA resulting in an increase in template, which could be used to produce more RNA4.9. This brings into question if the results are really due to a decrease in RNA4.9 or if it is disrupting the cis-elements within oriLyt which would result in a decrease in DNA replication and then a decrease in RNA4.9. The other issue with the kinetics of RNA4.9 expression is that some of the experiments were performed at 24 hrs (Fig. 3 panel a) when they have not established that RNA4.9 is expressed at significant levels by 24 hpi. Fig. 1A extrapolates the levels of RNA4.9 from 24 to 72 hpi and it’s not clear if RNA4.9 is an early transcript or expressed after the onset of DNA synthesis. The knockdown of RNA4.9 (Fig. 2 panel a) is at 48 hpi, but that is already after the onset of DNA replication at 24-36 hpi.

We apologize for not clearly defining RNA4.9 expression kinetic in the previous version of our manuscript. Our RNA-seq measurement (revised Figure 1a) clearly shows RNA4.9 is already significantly expressed at 5hpi (much before DNA replication starts). We now also added data showing RNA4.9 is expressed prior to DNA replication but its expression is significantly enhanced when DNA replication starts (new Figure 2a). Furthermore we added data illustrating RNA4.9 localization to the replication compartment is probably independent of DNA replication as we can detect it in clear nuclear foci prior to the onset of DNA replication (new Figure 5c) and when DNA replication is inhibited (new Figure 2b).

Importantly, besides figure 2, in which we started characterizing the effects of RNA4.9 KD, KD efficiency of RNA4.9 in all other experiments was measured in the presence of PFA so any effect we measure is independent of reduction in DNA template.

In regards to the CRISPR experiments (Fig. 2, Fig. 4 and Fig. 6), what was the percentage of HCMV genomes that carried the sgRNA induced RNA4.9 promoter mutation? The authors attempt to answer this question with the relative induced mutations shown in Figure S6 but it doesn’t give an absolute value to how many genomes have the mutation since it is a relative comparison between the specific gRNA to the control gRNA.

In order to estimate which mutations the CRISPR-Cas9 generated and their abundance we sequenced viral DNA that was extracted from cells that express sgRNAs that target RNA4.9 TSS site (sgRNA #3 and sgRNA #5). For both sgRNA #3 and sgRNA #5 in 40% of the viral DNA we sequenced we identified mutations specifically in the region that is targeted by the sgRNAs (these mutations were mostly deletions of 2-10bp). However, since we sequenced the DNA that was extracted at 96hpi it is likely that this mutation rate is an underestimate of the true abundance of the Cas9 introduced mutations (as it is likely mutated genomes are defective in their ability to replicate). The real-time PCR analysis of RNA4.9 expression shows that even in the presence of PFA RNA4.9 expression is downregulated by at least 90% (Figure 6 and Figure S3), this likely indicates that the targeting efficiency is very high.

This brings up another issue, the control elements within the origin and the RNA4.9 promoter. Promoters are often associated with activity of lytic origins, and the act of transcription is thought to help open the DNA for initiation of DNA synthesis or allow for assemble of the core enzymatic machinery. The SV40 promoter can functionally replace the HCMV oriLyt promoter, demonstrating that the act of transcription is an important process for oriLyt-dependent DNA replication. The RNA4.9 promoter may have additional functions beyond the expression of the RNA4.9 transcript, it may in fact be a control element within the origin for initiating. The authors have not ruled out the possibility that they are disrupting the function of the cis-elements in the origin. This is a very difficult issue to resolve but there is no evidence that the phenotype described is due to the disruption of the RNA4.9 promoter which causes a decrease in the expression of RNA4.9 or if the disruption of the RNA4.9 promoter is directly affecting the ability of the origin to function normally.

The reviewer is correct and in our experiments we cannot distinguish between the act of transcription and the function of the RNA itself. Still, the high G+C content within the 5' of RNA4.9 and the generation of the R-loop does suggest at least some properties of the RNA itself play a role in RNA4.9 function but it certainly doesn’t prove this. We now explicitly discuss these possibilities (lines 338-345).

The R-loop within the origin has previously been described. In addition to the RNA4.9 R-loop there are other RNAs that have been identified from the origin. This was first reported in Prichard et al. J Virol. 1998 Sep;72(9):6997-7004 “Identification of persistent RNA-DNA hybrid structures within the origin of replication of human cytomegalovirus”. Interestingly, the 5’ region of RNA4.9 is within the transient plasmid-based replication assay and maybe functional in that system as well. The viral protein UL84 was also shown to interact with the stem-loop structure at the 5’ region of the RNA-DNA hybrid in Colletti et al. J Virol. 2007 Jul;81(13):7077-85 “Human Cytomegalovirus UL84 Interacts with an RNA stem-loop sequence found within the RNA/DNA hybrid region of oriLyt”.

We thank the reviewer for bringing these papers to our attention. The RNA that were mapped by Prichard et al. (suggested to be 300 and 500 bases long) were mapped at the opposite orientation relative to RNA4.9. Recent RNA-seq using poly-A selection failed to capture these RNAs, but it remains possible these RNAs are not polyadenylated. If these RNAs are indeed expressed it is possible that there is a complex transcription pattern around the *oriLyt*, supporting its activity. This possibility is now discussed (lines 373-382). We also now added data that shows that UL84 recruitment does not seem to be effected by RNA4.9 KD (new Figure S4a).

Fig. 4 panel c and d. Based on the western blot it seems like there might also be a reduction in the amount of UL84 and UL44 protein in cells treated with the sgRNA3 and not a specific reduction in the ssDBP levels. The graph in panel d shows the ssDBP relative to GAPDH, what are the levels of UL84 and UL44 in the same graph. Additionally, with panel f what are the relative levels of early transcripts UL84 and UL44 compared to UL57?

As requested by the reviewer we now quantify the changes of UL44 and UL84 when we KD RNA4.9 (new Figure S4b). This analysis shows that in the presence of PFA there are no major changes in UL44 and UL84 proteins when we use RNA4.9-Cas9 KD whereas ssDBP expression is substantially reduced (Figure 4e). Furthermore, we added a new figure (Figure S3) in which we quantify the changes in expression of UL44 and UL84 transcripts. This new data supports our RNA-seq measurements that show that in the presence of PFA there are no major changes in early gene expression upon 4.9 RNA KD (data presented in table S1 )

Fig. 6 and 7. In figure 6 there is a decrease in RNA4.9 from the specific sgRNA and a decrease in UL57 RNA, while in Figure 7 there decrease in RNA4.9 in the OriShift mutant but the expression of UL57 is not significantly different in the WT and OriShift mutant (panel e). This difference in the amount of UL57 transcript detected (significantly different in cells where RNA4.9 is KD with Cas9 and sgRNA, but not significantly different in the oriShift mutant where RNA4.9 levels are reduced through genomic shifting) is not adequately discussion as to why there is an inconsistency in the method used to reduce RNA4.9 and the levels of UL57 that are reported.

In the places we used CRISPR-Cas9 to KD RNA4.9 expression, we usually obtain 90-95% KD efficiency which associate with up to 50% reduction in UL57 transcript. This reduction could be related to the actual cleavage of the viral DNA or to some coupling between UL57 transcription and the *oriLyt* activity. In the OriShift mutant (in which the origin was moved to a different location), we see a smaller reduction in RNA4.9 transcript (50%), which is expected since the promoter of RNA4.9 is likely fully embedded in the origin. In this settings, we observed no change in UL57 transcript level. Therefore, these differences can stem from trans effects mediated by RNA4.9 that depends on its levels or they can indicate that the CRISPR-Cas9 cleavage contributes to the subtle reduction in UL57 transcript levels.

Figure S2. The other compounding factor is if the CRISPRi protein, which binds to DNA to block transcription without the nuclease activity, could block the ability of the viral DNA initiation protein in a similar manner, and if that’s true then the results could be due to the blocking IE2 or UL84 at the origin in addition to blocking transcription.

To address this point we now added data showing UL84 levels and its recruitment to the replication compartment are not affected by RNA4.9 KD (Figure S4a). This observation provides further support that the viral replication defects we are measuring are indeed related to the reduction in RNA4.9 levels and the corresponding reduction in UL57 levels. This conclusion is further supported by the observation the over expression of UL57 alleviates the inhibition in viral DNA replication and growth in this setting

**Part III – Minor Issues: Editorial and Data Presentation Modifications**
Please use this section for editorial suggestions as well as relatively minor modifications of existing data that would enhance clarity.

Reviewer #1: 1) It would be helpful to include a second guide RNA (ie:sgRNA5) in at least panel F of figure 4
This data is presented in figure 6. sgRNA#5 in figure 6 is the sgRNA#5 that is used in the rest of the manuscript. This is now more explicitly stated in the text.

2) Does PFA treatment limit accumulation of ectopically expressed ssDBP flag in HCMV infected cells? This would be predicted to occur and is simple to test.

As requested by the reviewer we assessed the expression of ectopically expressed ssDBP in cells treated with PFA (revised Figure 5d and 5e). As seen in these figures adding PFA to infected cells had only a mild effect on ectopically expressed UL57-Flag protein. This result goes in line with our experiments showing that treating cells with PFA leads to reduction in ssDBP expression that was comparable to other viral early proteins (in control cells ssDBP expression was affected by PFA treatment to the same extent as other early proteins, Figure 4d). Since PFA blocks viral DNA replication by inhibiting the viral DNA polymerase , these results support the assumption that the initial steps of replication, which precede DNA synthesis, such as the *oriLyt* unwinding, are the ones that are coupled to ssDBP levels. This point is now explicitly discussed in the manuscript (lines 357-369)

Editorial comments to address in the text:
Line 199-200 revise to state,” We next tested the possibility that RNA4.9 KD affects ssDBP stability perhaps as an outcome of faulty unwinding of oriLyt. “

This has been revised

Line 205, replace “started” with “first observed”

This has been revised


Line 207-209: As written, this is somewhat confusing, whereas it is very clear in the figure 9 legend. I think the authors are combining too much in a single sentence and as a result may make it harder for the generalist reader to separate experimental findings from more interpretative speculation.

This sentence as been revised

“These results indicate that in the absence viral DNA replication, which may reflect the *oriLyt* unwinding, viral ssDBP levels are low."

Figure 5 and S5 legends: the authors measure DNA accumulation, which of course reflects DNA replication. The term “initiation”, however, reflects a discrete phase of the replication process that is inferred by the authors, and while likely correct, has not been demonstrated experimentally. Perhaps the term “initiation” could be removed from the legend and retained in the text in the speculative manner in which it is already used by the authors.

The reviewer is correct and these changes in the figure legend were incorporated.

Reviewer #2: (No Response)

Reviewer #3: Minor points:
Page 4 line 66-68: In regards to the reports of viral lncRNAs, Noriega et al. J Virol. 2014 Aug;88(16):9391-405 “Human cytomegalovirus modulates monocyte-mediated innate immune responses during short-term experimental latency in vitro” has also identified HCMV lncRNAs during latency.

This reference was added to the manuscript.
